# Characteristics of the gut microbiota and the effect of *Bifidobacterium* in very early-onset inflammatory bowel disease patients with IL10RA mutations

**DOI:** 10.3389/fmicb.2024.1479779

**Published:** 2024-12-02

**Authors:** Xu Xu, Yuanqi Gao, Yuan Xiao, Yi Yu, Jiebin Huang, Wen Su, Na Li, Chundi Xu, Shenshen Gao, Xinqiong Wang

**Affiliations:** ^1^Department of Pediatrics, Ruijin Hospital, Shanghai Jiao Tong University School of Medicine, Shanghai, China; ^2^Department of Tropical Diseases, The Second Affiliated Hospital of Hainan Medical University, Key Laboratory of Tropical Translational Medicine of Ministry of Education, NHC Key Laboratory of Tropical Disease Control, School of Tropical Medicine, Hainan Medical University, Haikou, Hainan, China; ^3^Clinical Research and Development Center of Shanghai Municipal Hospitals, Shanghai Shenkang Hospital Development Center, Shanghai, China

**Keywords:** *Bifidobacterium*, dysbacteriosis, gut microbiota, IL10RA mutant, very early-onset IBD

## Abstract

Very early-onset inflammatory bowel disease (VEO-IBD) is a distinct subtype of inflammatory bowel disease (IBD) characterized by onset before the age of 6 years, and patients often exhibit more severe clinical features. Interleukin 10 receptor alpha (IL10RA) is a hotspot mutation in the Chinese population and is associated with a poor prognosis closely linked to the onset of IBD. However, limited knowledge exists regarding how the IL10RA mutation influences the host microbiota and its role in disease development. We employed 16S rRNA sequencing to conduct a comprehensive assessment of microbial changes in different types of IBD, employed database to thoroughly examine the influence of *Bifidobacterium* in IBD and to demonstrate a potential positive effect exerted by Bifidobacterium breve M16V (M16V) through a mouse model. The study demonstrated a significant reduction in the abundance and diversity of the gut microbiota among children with IL10RA mutations compared to those with late-onset pediatric IBD and nonmutated VEO-IBD. Furthermore, the analysis identified genera capable of distinguishing between various types of IBD, with the genus *Bifidobacterium* emerging as a potential standalone diagnostic indicator and *Bifidobacterium* may also be involved in related pathways that influence the progression of IBD, such as the biosynthesis of amino acids and inflammation-related pathways. This study corroborated the efficacy of *Bifidobacterium* in alleviating intestinal inflammation. The impact of IL10RA mutations on VEO-IBD may be mediated by alterations in microbes. M16V demonstrates efficacy in alleviating colitis and holds promise as a novel microbial therapy.

## 1 Introduction

Inflammatory bowel diseases (IBDs), which encompass Crohn's disease, ulcerative colitis, and an unspecified subtype, are chronic immune-mediated intestinal disorders (Agrawal et al., 2021). Global epidemiologic trends demonstrate an increasing incidence and prevalence of IBD (Ng et al., 2017), which is influenced by environmental, genetic, immunological, and intestinal microecological factors (Abbas-Egbariya et al., 2022; Khalili et al., 2018; Peloquin et al., 2016). However, the exact pathogenic mechanism remains elusive. Pediatric-onset IBD (PIBD), which affects children, presents with distinct challenges, with differences in growth, development, and clinical severity compared to adult-onset cases (Oliveira and Monteiro, 2017). Recent advancements in sequencing technology have demonstrated a unique phenotype in children under 6 years of age, which is known as very early onset IBD (VEO-IBD) and which is often characterized by severe clinical manifestations (Hall and de Zoeten, 2023; Uhlig et al., 2014). Genetic factors are strongly associated with VEO-IBD, with 80 single gene mutations identified to date (Sharifinejad et al., 2022; Uhlig et al., 2021). IL10, which is crucial for intestinal homeostasis, offers protection from intestinal injury and limits the development of colitis (Zegarra Ruiz et al., 2022). Mutations in IL10 and its receptor gene are clearly associated with VEO-IBD. A large cohort study suggests that a lack of IL10 or IL10 receptors can lead to severe colitis in newborns, which can be life-threatening and accompanied by multiple extraintestinal manifestations (Huang et al., 2017).

Although genetic factors are significant in the development of VEO-IBD, the incidence and prevalence of this condition are escalating, indicating an influence from environmental and microbial factors on its development (Kuenzig et al., 2022). Numerous studies have established a significant correlation between the gut microbiota and IBD (Lloyd-Price et al., 2019; Ekstedt et al., 2024), with some studies suggesting that the gut microbiota may serve as one of the primary driving factors (Nishida et al., 2017; Kostic et al., 2014). During the first few years of life, microbial communities exhibit inherent characteristics of reduced diversity, immaturity, and rapid fluctuations, which are closely aligned with the concurrent development of the immune system (Subramanian et al., 2015; Bokulich et al., 2016). Research has demonstrated the multifaceted influence of the intestinal microbiota on IBD across various age groups. This influence involves the presence of pathogenic microbial communities and immune dysregulation directed against commensal microbiota, ultimately resulting in dysbiosis within the microbial community structure (Haberman et al., 2014; Olbjørn et al., 2019). Previous studies have demonstrated that both germ-free mice and germ-free IL-10 knockout mice fail to develop colitis, thereby confirming the role of the microbiota in the initiation and progression of inflammation. Additionally, the transplantation of feces from colitis mice to healthy mice can cause healthy mice to also develop colitis (Arnauts et al., 2022; Wang et al., 2019). But there are few studies related to gut microbes in children with either VEO-IBD or IL10 receptor-deficient IBD, as well as its underlying mechanisms. This study investigated the relationship between microbial ecological imbalance and intestinal inflammation in children with VEO-IBD and IL10RA mutations in IBD patients. Changes in the gut microbiota were analyzed to explore the relationship between microbial ecological imbalance and intestinal inflammation in children with IBD. Additionally, beneficial bacteria that may play a positive role in future research are identified to provide new therapeutic options.

## 2 Materials and methods

### 2.1 Diagnostic and inclusion criteria

In this study, we recruited two PIBD cohorts, VEO-IBD and late-onset IBD (LO-IBD), from Ruijin Hospital, Shanghai, between January 1, 2019, and December 31, 2022. We also recruited a group of healthy control (HC) subjects who were carefully matched by age across the VEO-IBD group. It should be noted that all participants and guardians who were enrolled provided informed consent. The enrollment followed the specific inclusion and exclusion criteria, which are provided in the follow.

The inclusion criteria included: (1) Participants must be aged between 0 and 18 to be eligible. (2) Each patient diagnosed with IBD underwent a complete physical examination, gastrointestinal endoscopy, pathology and radiological imaging, and the diagnosis was ultimately confirmed by three or more pediatricians. The diagnosis was made according to the Porto criteria (IBD Working Group of the European Society for Paediatric Gastroenterology, Hepatology and Nutrition, 2005). (3) The control group was a healthy control population. (4) The participants and guardians were willing to cooperate in the collection of fecal samples and basic and clinical information. The exclusion criteria include: (1) medication history of antibiotics, probiotics, and immunosuppressants within 1 month before enrollment; (2) significant changes in dietary habits within 6 months before enrollment; (3) inability to cooperate or unwillingness to cooperate with this study. None of the children received any IBD-related treatment. The protocol was approved by the Institutional Review Board of Ruijin Hospital, Shanghai Jiao Tong University School of Medicine.

VEO-IBD is children with IBD between the ages of 0 and 6-years-old; LO-IBD is children with IBD between the ages of 6 and 18-years-old. Children diagnosed with VEO-IBD underwent further categorization through whole-exome sequencing into IL10RA-mutated and non-IL10RA-mutated groups.

Clinical data pertaining to the participants, including age, sex, weight, height, Body Mass Index (BMI), and laboratory findings [including C-reactive protein (CRP) and erythrocyte sedimentation rate (ESR)], were collected. We calculated height and weight Standard Deviation Score (SDS) based on age and sex, using survey data on the physical development of children in nine cities in China after taking height and weight measurements for all participants (Capital Institute of Pediatrics, The Coordinating Study Group of Nine Cities on the Physical Growth and Development of Children, 2018). Symptoms of illness were extracted from the medical records. Disease activity in children was scored accordingly after sample collection by using the Children's Inflammatory Bowel Disease Activity Index, which includes the Pediatric Crohn's Disease Activity Index (PCDAI) and Pediatric Ulcerative Colitis Activity Index (PUCAI) (Hyams et al., 1992; Turner et al., 2009).

### 2.2 Sample handling and 16S rRNA gene sequencing

All participants are required to provide a minimum of 3 g of stool sample. These samples were collected from the children's daily excretions, selected from the mid-ribs of the samples, and ensured that the samples selected did not contain urine. Samples are to be collected in a sterile specimen collector provided by the investigator in advance. After collection, the samples must be promptly transferred to a −80°C ultra-low temperature cryogenic freezer for storage within 4 h. The collected samples were subjected to DNA extraction by using an E.Z.N.A. kit. The DNA concentration and purity were determined by using a NanoDrop 2000 spectrophotometer, and DNA quality was verified via 1% agarose gel electrophoresis. The concentration of all of the samples was kept above 50 μg/μl, and 10 ng of extracted DNA was split for 16S rRNA.

Total DNA extraction and PCR amplification were performed according to a previously described protocol (Wang et al., 2021). Briefly, an E.Z.N.A.^®^ soil kit (Omega Biotek, Norcross, GA, USA) was used for DNA extraction. The primers 338F (5′-ACTCCTACGGGGAGGCAGCAG-3′) and 806R (5′-GGACTACHVGGGGTWTCTAAT-3′) were used for PCR amplification of the V3–V4 variable region. Subsequently, Illumina MiSeq platform (Illumina, San Diego, USA) standard operating procedures were followed for analysis, and the purified amplified fragments were utilized to construct PE 2^*^300 libraries. Sequencing was conducted using the Illumina MiSeq PE300 platform (Shanghai Meiji Biomedical Technology Co., Ltd.).

### 2.3 Bioinformatics analysis for interaction between host transcriptome and microbiota

We gathered 78 RNA-seq datasets and related 16S rRNA data to investigate the functions of *Bifidobacterium*. The RNA-seq database for pediatric IBD (project accession number E-MTAB-5464) was obtained from the European Bioinformatics Institute (EBI) at https://www.ebi.ac.uk/gxa/experiments/E-MTAB-5464. Complementary 16S rRNA sequencing data from the same research study were acquired from the EBI, with the study identification number PRJEB6663. To discern variations in the microbial expression profiles, we employed *t*-tests for statistical inference. The diagnostic efficacy of *Bifidobacterium* populations was rigorously appraised utilizing the framework of ROC curves. Subsequently, the identification and functional interpretation of differentially expressed genes, as well as the enrichment analysis of Kyoto Encyclopedia of Genes and Genomes (KEGG) pathways, were conducted employing the robust computational tools provided by the R programming environment, specifically the limma and clusterProfiler packages.

### 2.4 Processing of mouse model samples and measurement of inflammatory factors

We administered drinking water to three groups of 8-week-old SPF wild-type C57BL/6 mice. *Bifidobacterium* breve M16V (M16V), which is a classical *Bifidobacterium*, was selected as our primary choice. The specific grouping scheme comprised the normal drinking water group, the 3% DSS-administered group, and the 3% DSS + M16V-administered group, with M16V administered at a dose of 2 × 10^8^ CFU/day. The modeling period lasted for 7 days, and on the 8th day, samples were collected and processed from the three groups of mice. Weight changes, colon length, and disease activity index (DAI) scores were assessed. Colon tissues were harvested for pathological histological section analysis, followed by tissue scoring. The collected data were subsequently subjected to statistical analysis. Colon samples were collected from the mouse model, and the colon protein was obtained as previously described (Wang et al., 2023). The total protein concentration was quantified by using a BCA assay (Thermo Fisher Scientific, MA, USA). All of the samples were diluted to a uniform concentration for further examination. Using a 10-plex magnetic bead-based immunoassay kit (Bio-Rad, CA, USA), the following cytokines were quantified: IL-10, IL-12, CXCL1, IFN-gamma, TNF-alpha, IL-1 beta, IL-2, IL-4, IL-5, and IL-6. The Bio-Plex 200 system was utilized for the analysis of the kit according to the manufacturer's directions. The results were analyzed by using a Luminex instrument.

### 2.5 Analysis of data

Categorical variables are displayed as frequencies along with their respective percentages. Continuous variables are expressed as the mean accompanied by the standard deviation. Fisher's exact test is suitable for cases with sample size *N* < 40 or theoretical frequency *t* < 1. To compare continuous variables between two groups, the Mann–Whitney *U*-test was utilized. Spearman correlation analysis was conducted to evaluate the correlation between variables. Multiple tests were adjusted by using the conservative Bonferroni correction. A two-tailed *P*-value of < 0.05 was considered indicative of statistical significance. In the bioinformatics analyses, alpha diversity metrics and beta diversity were computed by using bray curtis distances and visualized through principal coordinate analysis (PCoA) by using the open-access online tool known as the Majorbio Platform. The comparison between the two samples was conducted using the Kruskal-Wallis test for significance assessment. Correlation analyses and Receiver Operating Characteristic (ROC) analyses were conducted by using the heatmap package in R, the pROC package, and the ggplot2 package to evaluate the diagnostic value of the identified indicators (Robin et al., 2011).

## 3 Results

### 3.1 Clinical data characterizing the patients and the controls

A total of 32 children diagnosed with VEO-IBD, 32 children with LO-IBD, and 15 healthy children were enrolled in the study. We conducted exome sequencing to identify mutation information for all VEO-IBD children. IL10RA mutations were identified in 16 patients, as detailed in Table 1. In line with our study's objectives, we provided detailed clinical information for children across the IL10RA mutation, non-IL10RA mutation, LO-IBD and HC groups. Specifically, Table 2 presents the comprehensive clinical data, symptoms, and laboratory parameters for all three IBD groups and the HC controls. Our analysis demonstrated that children with IL10RA mutations were diagnosed at a significantly younger age than those without mutations (*P* = 0.0012). Additionally, children with IL10RA mutations exhibited markedly higher levels of inflammatory markers, including CRP and ESR, compared to the non-mutated group, with significant disparities in PCUAI/PCDAI scores. Clinical symptoms, including perianal lesions and fever, were more prevalent among children with IL10RA mutations than among those in the nonmutated group. These findings collectively suggest that children harboring the IL10RA mutation exhibit greater disease activity and severity than those without the mutation.

**Table 1:** Mutation information for children with VEO-IBD^*^.

**Patient**	**Gene**	**Variant (allele 1)**	**ACMG (P/LP)**	**Variant (allele 2)**	**ACMG (P/LP)**
1	IL10RA	c.301 C>T	P	c.301 C>T	P
2	IL10RA	c.299 T>G	P	c.301 C>T	P
3	IL10RA	c.191 A>G	LP	c.537 G>A	P
4	IL10RA	c.493 C>T	P	c.301 C>T	p
5	IL10RA	c.301 C>T	P	c.537 G>A	P
6	IL10RA	c.350 G>A	P	c.493 C>T	P
7	IL10RA	c.301 C>T	P	c.436delC	P
8	IL10RA	c.301 C>T	P	c.537 G>A	P
9	IL10RA	c.421 G>A	P	c.537 G>A	P
10	IL10RA	c.109G>T	LP	c.301 C>T	P
11	IL10RA	c.301 C>T	P	c.537 G>A	P
12	IL10RA	c.302G>A	LP	c.349C>T	P
13	IL10RA	c.299 T>G	P	c.569T>G	LP
14	IL10RA	c.537 G>A	P	c.537 G>A	P
15	IL10RA	c.299 T>G	P	c.299 T>G	P
16	IL10RA	c.299 T>G	P	c.299 T>G	P

^*^Where the gene information not shown is for children with non-mutated VEO-IBD.

**Table 2:** Clinical data characterizing the patients and the controls.

	**VEO-IBD**			**LO-IBD**	**P2**	**HC**	**P3**
	**IL10RA mutation**	**Non-IL10RA mutation**	**P1**				
Samples	16	16	NA	32	NA	15	NA
Age (year)	1.25 ± 0.74	2.75 ± 1.57	0.0012	12.7 ± 21.9	< 0.001	2.1 ± 1.51	0.5189
Sex (boy/girl)	10/6	10/6	>0.999	20/12	>0.999	5/10	>0.999
Weight-SDS	−0.66 ± 1.50	0.19 ± 2.49	0.4677	−1.03 ± 0.88	0.1357	NA	NA
Height-SDS	−0.86 ± 2.90	0.56 ± 3.37	0.2242	−1.13 ± 1.42	0.3142	NA	NA
BMI (kg/m^2^)	15.06 ± 1.98	15.48 ± 1.54	0.3414	16.15 ± 2.43	0.1606	NA	NA
**Disease activity**							
CRP (mg/L)	46.60 ± 27.33	6.92 ± 9.32	< 0.0001	12.43 ± 29.21	0.0068	NA	NA
PCDAI/PUCAI score	40.44 ± 16.26	15 ± 10.21	< 0.0001	35.39 ± 14.62	0.0655	NA	NA
ESR	19.38 ± 14.32	13.06 ± 15.56	0.0070	15.13 ± 11.79	0.4085	NA	NA
**Clinical manifestation**							
Stomach	12.50%	31.25%	0.3944	75.00%	< 0.001	NA	NA
Diarrhea	93.75%	68.75%	0.1719	50.00%	0.0169	NA	NA
Hematochezia	87.50%	68.75%	0.3944	25.00%	< 0.001	NA	NA
Perianal lesions	68.75%	12.50%	0.0032	12.50%	0.0219	NA	NA
Fever	75.00%	37.50%	0.0076	47.00%	0.8029	NA	NA

P1 value for the comparison between IL10RA mutation group and non-IL10RA mutation group, P2 value for the comparison between VEO-IBD group and LO-IBD group, P3 value for the comparison between VEO-IBD group and HC group, SDS standard deviation score based on age and sex. CRP, C-reactive protein; PCDAI, pediatric Crohn's disease activity index; PUCAI, pediatric Ulcerative Colitis activity index; ESR, erythrocyte sedimentation Rate.

### 3.2 Characterization of gut microbial diversity

**Figure 1:**
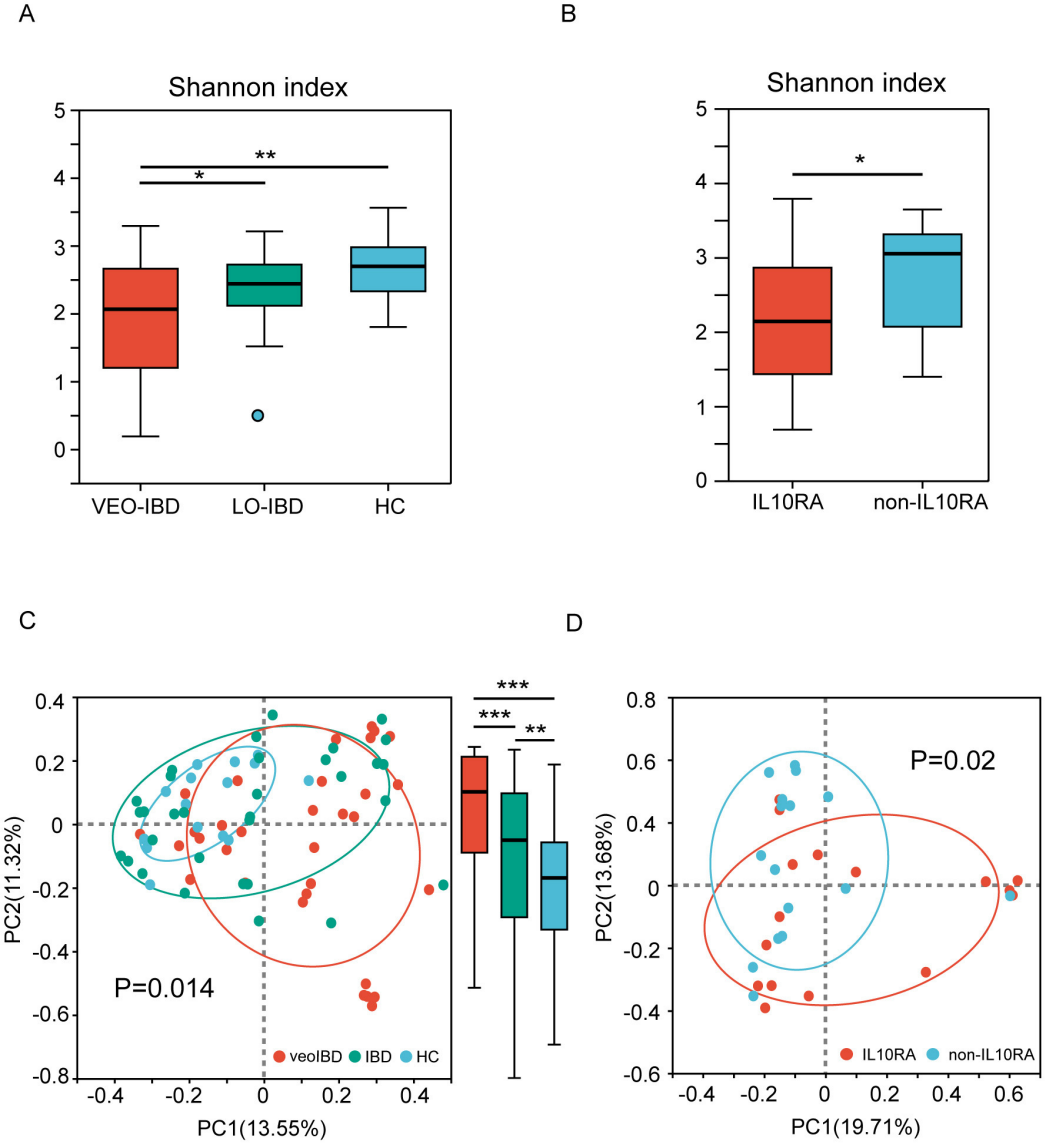
Alpha and beta diversity of the gut microbiota. **(A)** Shannon index of gut microbes in three subgroups (VEO-IBD, LO-IBD, and HC). **(B)** Shannon indices of gut microbes in two subgroups (IL10RA-mutant VEO-IBD and non-IL10RA-mutant VEO-IBD). **(C)** PCoA plots of bray curtis distances of the gut microbial community structure in the three subgroups. **(D)** PCoA plots of bray curtis distances of the gut microbial community structure in the two subgroups. VEO-IBD, Very early-onset IBD group; LO-IBD, late-onset IBD group; HC, Healthy children group; non-IL10RA, non-IL10RA mutant VEO-IBD. *P < 0.05, **P < 0.01, ***P < 0.001.

Upon analyzing the gut microbiota in stool samples from children in comparison with the HC group, we observed reduced gut microbial alpha and beta diversity in both the VEO-IBD and LO-IBD groups. The Shannon indices were 1.94 ± 0.90 for VEO-IBD, 2.35 ± 0.54 for LO-IBD, and 2.66 ± 0.50 for HC children (Figure 1A). Furthermore, the microbial diversity in children with IL10RA mutations was lower than that in non-IL10RA mutations, showing Shannon indices of 1.60 ± 0.90 for IL10RA mutations and 2.29 ± 0.79 for non-IL10RA mutations, respectively (Figure 1B).

Analysis of beta diversity, which was measured as the bray curtis distance of the operational taxonomic unit (OTU) community structure, demonstrated that the microbiota of the HCs was more tightly clustered, thus indicating greater similarity in their microbial profiles. Conversely, microbial profiles in children with IBD showed alterations across all of the samples, particularly in VEO-IBD. Moreover, we found significant *P*-values within the groups as well, with the *P*-value between the VEO-IBD group and the IBD group being 1.23e-13, the *P*-value between the IBD group and the HC group being 0.004382, and the *P*-value between the VEO-IBD group and the HC group being 9.328e-13 (Figure 1C). The microbiota of children with IL10RA mutations showed greater heterogeneity compared to that of non-mutated VEO-IBD patients (Figure 1D).

### 3.3 Changing relationships of microbial structural groups

**Figure 2:**
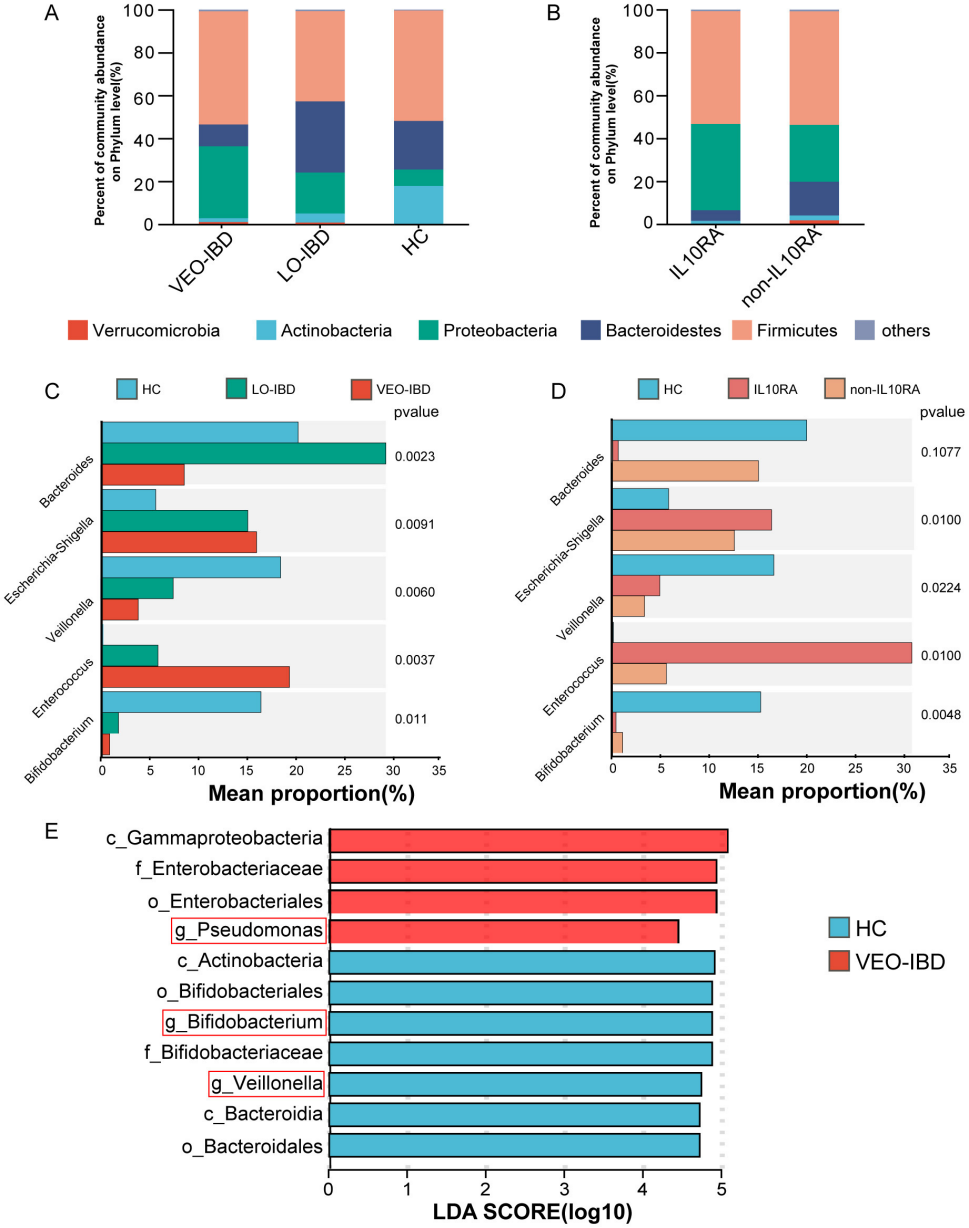
Structure and relative abundance analysis of the gut microbiota. **(A)** Percent community abundance at the phylum level of the gut microbiota in the three groups. **(B)** Percent community abundance at the phylum level of the gut microbiota in the two sub-groups. **(C)** Comparison of the five most variable microbes of gut microorganisms in the HC, LO-IBD and VEO-IBD subgroups. **(D)** Comparison of the five most variable microbes of gut microorganisms in the HC, non-IL10RA and IL10RA subgroups. **(E)** The LDA results were compared between VEO-IBD patients and HCs (LDA score > 4). VEO-IBD, Very early-onset IBD group; LO-IBD, late-onset IBD group; HC, Healthy Children group; non-IL10RA, non-IL10RA mutant VEO-IBD.

In the analysis of the fecal microbiota, we observed a rapidly expanding proportion of *Proteobacteria* and a significant decrease in *Actinobacteria* in children with IBD compared to those in the HCs (Figure 2A). The percentage of *Proteobacteria* increased, while the percentage of *Actinobacteria* decreased in the IL10RA-mutant VEO-IBD group (Figure 2B). These findings indicated that changes in *Proteobacteria* and *Actinobacteria* may be closely associated with IBD. At the genus level, *Bacteroides, Escherichia-Shigella, Veillonella, Enterococcus* and *Bifidobacterium* were the five genera with the greatest changes observed among the groups. The proportion of *Bifidobacterium*, which is an *Actinobacteria*, was markedly reduced (Figure 2C). Further comparison between the two VEO-IBD groups and the healthy group revealed it was found that *Bifidobacterium* was less prevalent in the IL10RA group compared to the non-IL10RA group (Figure 2D). We focused our analysis at the genus level and discovered through linear discriminant analysis (LDA) that the genus *Bifidobacterium* was significantly underrepresented in the VEO-IBD group, as illustrated in Figure 2E. These findings suggest that *Bifidobacterium* could be a significant factor in the development or progression of IBD.

### 3.4 Correlation of clinical data with samples and the diagnostic value of ROC curves

**Figure 3:**
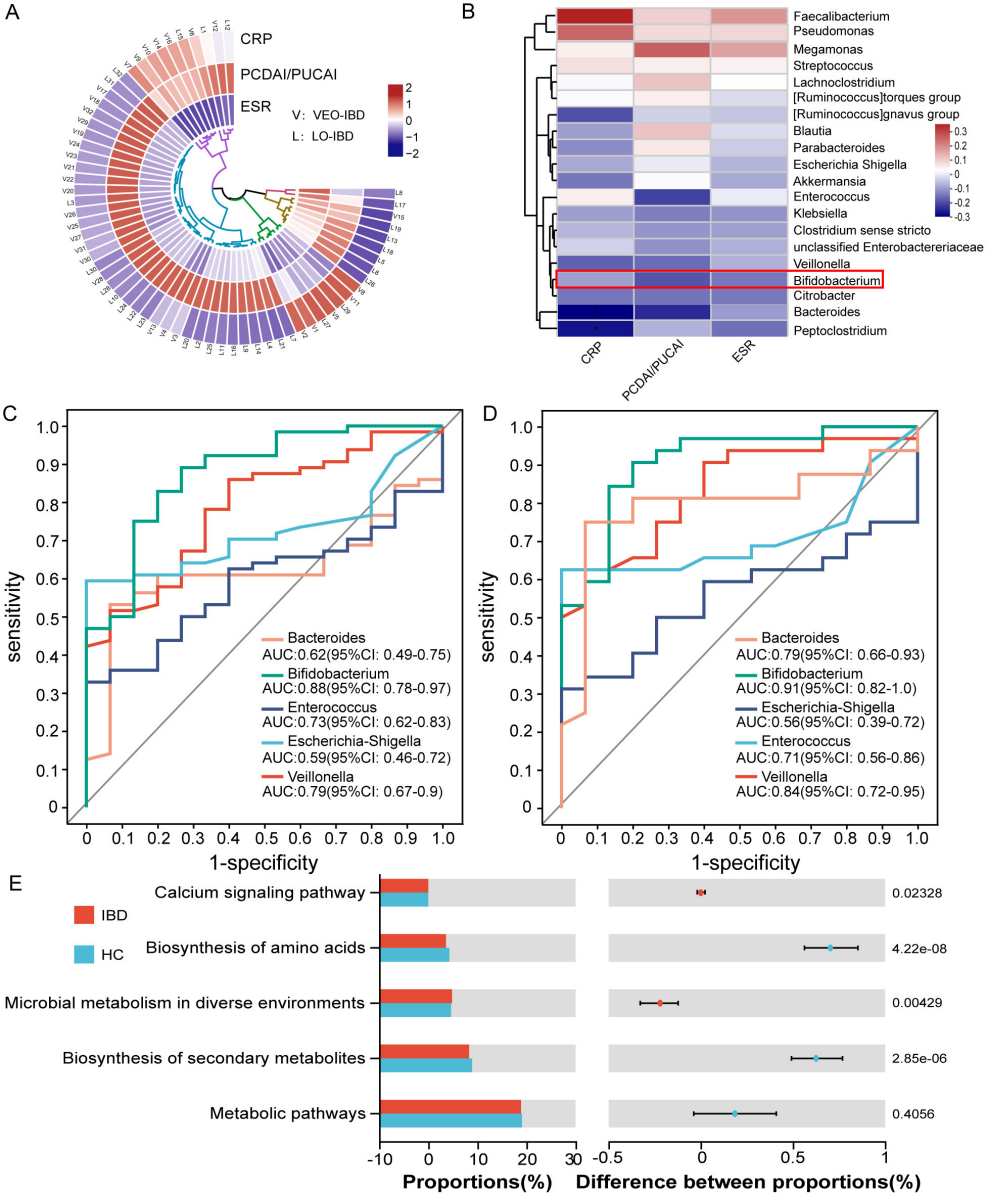
Relationships and functional predictions between the microbiota and ROC curve analysis of diagnostic indicators. **(A)** Correlation analysis between clinical data of children with VEO-IBD and those of children with LO-IBD. **(B)** Correlation analysis between clinical data and the top 20 genera. **(C)** ROC curves of the model were evaluated by using five major genera separately for identifying IBD patients and HC patients, optimal cutoff value for *Bifidobacterium* is 0.0028. **(D)** ROC curves of the model were evaluated by using five major genera separately for identifying VEO-IBD patients and HC patients, optimal cutoff value for *Bifidobacterium* is 0.0076. **(E)** Functional pre-diction analysis between children with IBD and HCs. ROC, Receiver Operating Characteristic; VEO-IBD, Very early-onset IBD group; HC, Healthy Children group.

By analyzing the correlation among clinical data, microbes, and children diagnosed with IBD, we observed a stronger correlation between PCDAI/PUCAI scores and the type of IBD in the children. Most children with VEO-IBD exhibited higher scores than children with LO-IBD (Figure 3A). Further examination of the correlation between microbes and clinical data demonstrated a predominantly negative correlation between inflammatory markers (CRP, ESR and DAI) and *Bifidobacterium*, which aligned with our expectations and suggested a likelihood of dysbiosis among children with IBD (Figure 3B). ROC curve analysis was employed to further evaluate the differential microbes and explore their diagnostic value for IBD. The five most abundant genera (*Bacteroides, Escherichia-Shigella, Veillonella, Enterococcus* and *Bifidobacterium*) were included in the optimal classification model. We isolated the genera to pinpoint the most effective diagnostic marker through binomial logistic regression analysis. *Bifidobacterium* showed considerable diagnostic potential in both models (IBD group and HC group, VEO-IBD group and HC group). The area under the curve (AUC) was 0.88 for the IBD model (95% CI: 0.78–0.97, *P* = 0.001, cutoff value: 0.0028) and 0.91 for the VEO-IBD model (95% CI: 0.82–1.0, *P* = 0.001, cutoff value: 0.0076; Figures 3C, D). This suggests that the *Bifidobacterium* genus could be a valuable microbial marker for distinguishing in diagnostic models. In addition, an additional functional prediction analysis, performed on the microbial variations within our cohort, suggests that *Bifidobacterium* may contribute to the pathogenesis of IBD primarily through its impact on some metabolism-related pathways (Figure 3E). The result suggests that *Bifidobacterium* may be involved in disease progression by influencing metabolic pathways or metabolites, and this finding is supported by other studies (Marteau et al., 2001).

### 3.5 Multi-omics analysis reveals the alleviating effects of *Bifidobacterium* on IBD

**Figure 4:**
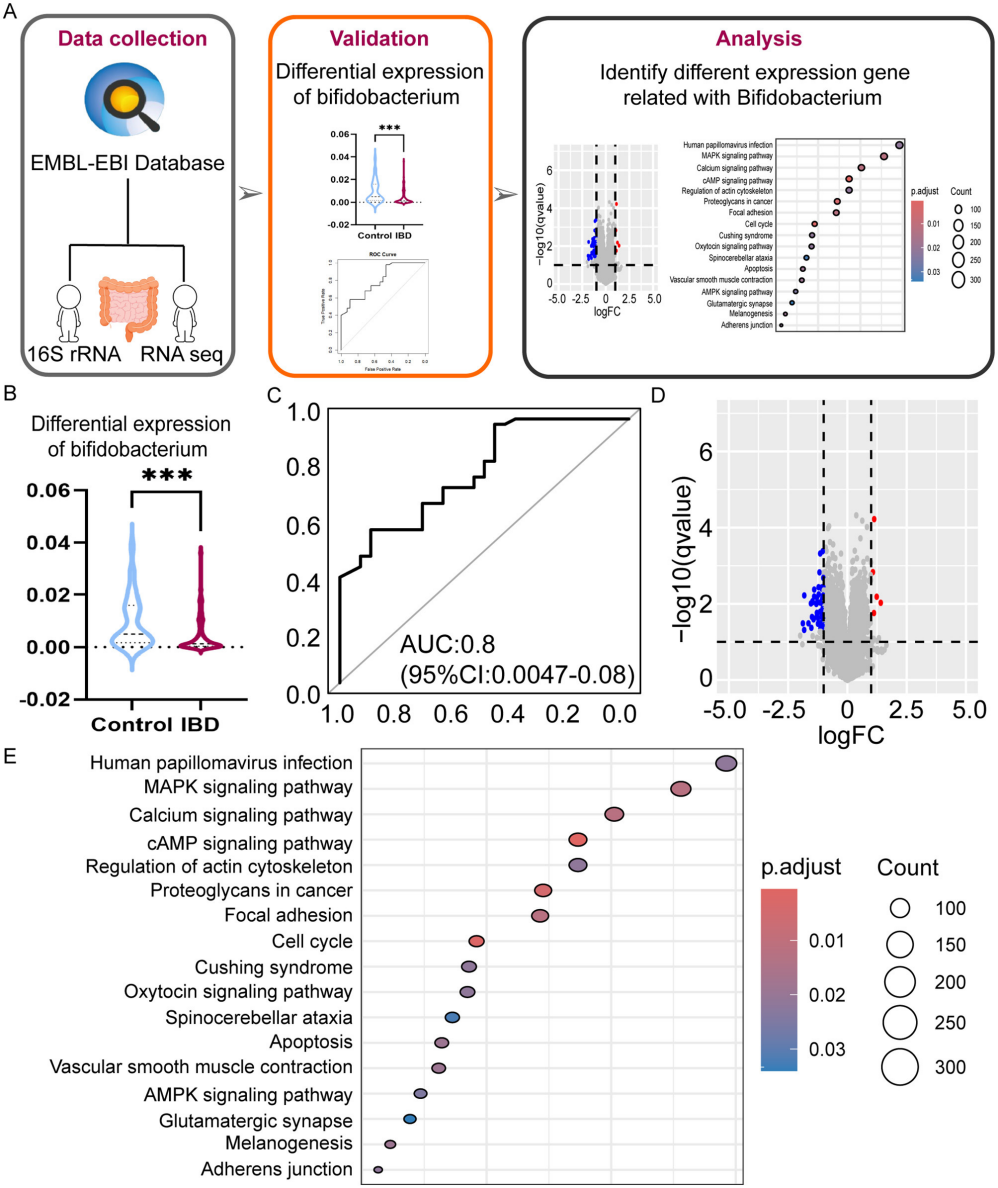
Multi-omics analysis reveals the role of *Bifidobacterium* on IBD. **(A)** The workflow of bioinformation analysis in the study. **(B)** Differences in *Bifidobacterium* expression in the two groups violin plots. **(C)** ROC curves were evaluated by using for identifying IBD patients and HC patients, optimal cutoff value for *Bifidobacterium* is 0.0025. **(D)** Differential gene volcano plots for different levels of expression in *Bifidobacterium*. **(E)** Bubble diagram of differential gene pathways with different expression levels in *Bifidobacterium*. ***P < 0.001.

A total of 78 RNA sequencing datasets were obtained after stringent filtration and control for subsequent analysis. The collated differential gene dataset is presented in Table 3. The detailed procedure of the study is shown in Figure 4A. We confirmed the significance of the difference in *Bifidobacterium* expression between PIBD and the control group (Figure 4B). Analyzing the performance curves of the subjects also demonstrated diagnostic significance, boasting an AUC of 0.8 (95% CI: 0.0047–0.008, cutoff value: 0.0025; Figure 4C). A set was downloaded from the European Bioinformatics Institute (EBI) database and analyzed to identify the differentially expressed genes (|logFC|> 1 and adjust *P* < 0.05). Visual mapping of the differential genes also revealed the presence of up-regulated genes (label in red) and down-regulated genes (label in blue) and identified *Bifidobacterium* in the KEGG pathway, which may be involved in the disease process primarily through its involvement in certain inflammatory pathways and calcium signaling pathways (Figures 4D, E). Previous studies have precisely shown that the calcium pathway is closely related to the intestinal mucosal barrier, and damage to the intestinal mucosal barrier induces colitis, thus further highlighting the close association between microbes and colitis (Engevik et al., 2019).

**Table 3:** Differentially expressed gene matrix.

**Gene name**	**Log fold change**	**Possession adjusted**	**Regulation**
MIR4429	1.130068	5.98E-05	Up
NEDD1	−1.04241	0.000416	Down
GLS	−1.14737	0.000478	Down
RELL1	1.075475	0.001472	Up
SLC6A18	−1.17724	0.001491	Down
SMAD9	−1.01755	0.002078	Down
ETFDH	−1.21429	0.003668	Down
KLC4	−1.07548	0.003686	Down
SEPTIN14	−1.01302	0.00371	Down
ZCWPW2	−1.03986	0.003769	Down
PPP1R14C	−1.07689	0.004016	Down
CCDC152	−1.39869	0.004222	Down
TNC	−1.20926	0.00565	Down
JAKMIP3	−1.81906	0.006016	Down
FAM227B	−1.02448	0.006039	Down
PLOD3	−1.4008	0.00643	Down
SURF1	1.235621	0.00652	Up
TMPRSS11E	−1.28506	0.006809	Down
TMEM14C	−1.06196	0.007438	Down
STC1	−1.04183	0.007731	Down
LHFPL2	−1.47191	0.008567	Down
MIR410	−1.47031	0.009112	Down
SCUBE1	−1.28033	0.009157	Down
TRIM46	−1.15234	0.009232	Down
MIR1296	1.406179	0.009404	Up
ANKRD30A	−1.5378	0.009776	Down
MAVS	−1.3096	0.010101	Down
ITPRIPL1	−1.02066	0.010203	Down
CHRDL2	−1.04741	0.010297	Down
ZNF827	−1.05006	0.013812	Down
TMEM79	−1.0584	0.015136	Down
TLCD4	−1.04782	0.015305	Down
RNF43	−1.14717	0.016139	Down
NOS2	−1.28675	0.017258	Down
LUM	1.120501	0.017446	Up
ABCA6	−1.08074	0.019595	Down
NEXN	−1.28313	0.020055	Down
FBLIM1	−1.04548	0.020159	Down
PBX3	−1.08509	0.021052	Down
ACRV1	−1.49272	0.022134	Down
OLFM4	−1.06287	0.022909	Down
CNKSR3	−1.24027	0.023759	Down
PEX11A	−1.43392	0.024272	Down
TAC1	−1.4266	0.025547	Down
CA7	−1.00543	0.026392	Down
SPATA16	−1.07546	0.030295	Down
LATS2	−1.64178	0.032524	Down
SCAPER	−1.87843	0.032564	Down
PYURF	−1.16489	0.033367	Down
ARRDC1	−1.24227	0.03338	Down
TPSG1	−1.00867	0.036535	Down
RND2	−1.03246	0.038925	Down
SPMIP9	−1.52054	0.042662	Down
KRT16	−1.00303	0.044288	Down
MIR3921	−1.13894	0.04429	Down
HMG20A	−1.82247	0.048526	Down

### 3.6 The role of *Bifidobacterium* M16V in mouse models

**Figure 5:**
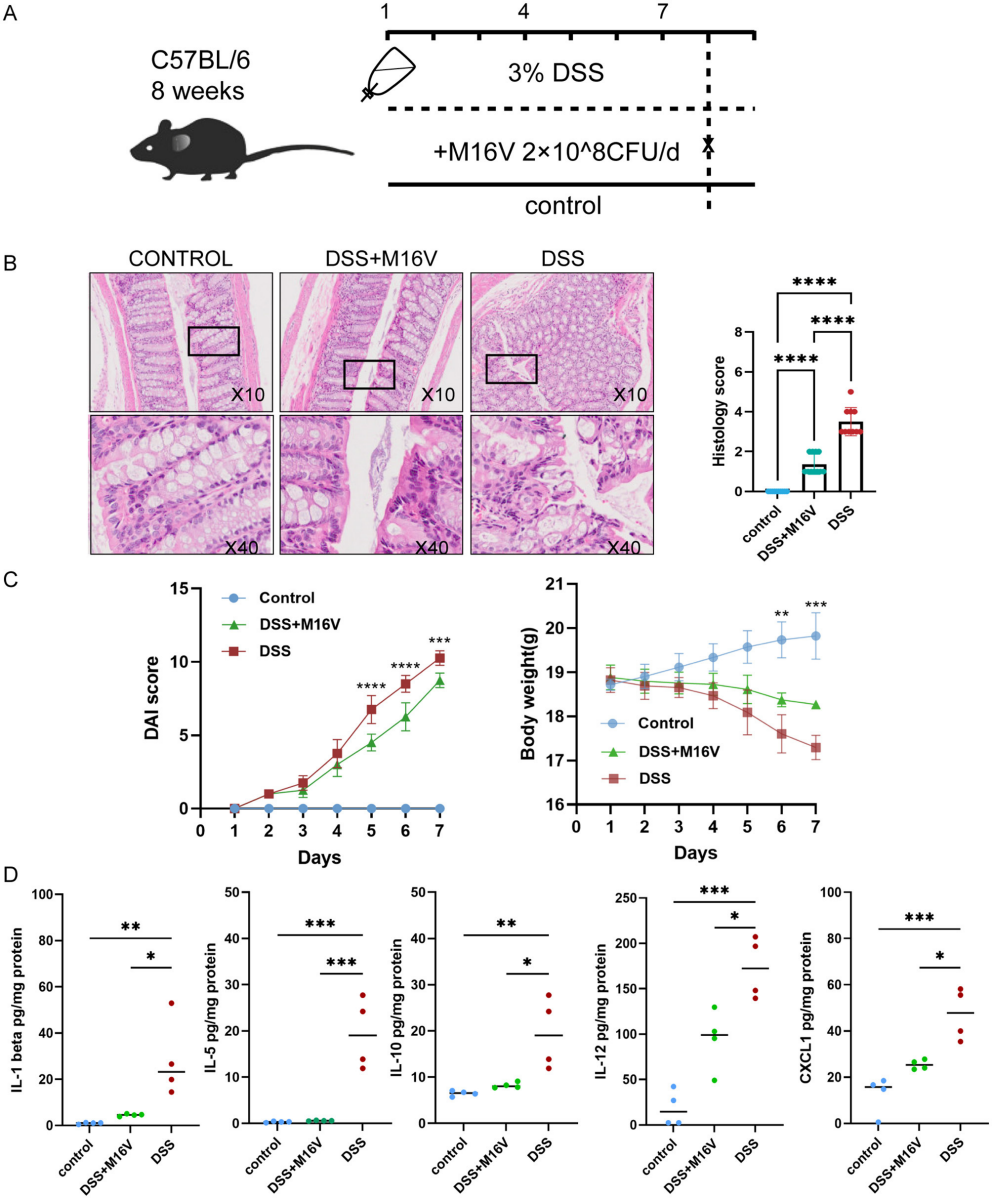
Effect of *Bifidobacterium* on DSS-induced mouse models and Comparison of the levels of five inflammatory factors between three groups. **(A)** Establishment and treatment of mouse models. **(B)** Bar graph representation of histopathological samples of mouse colon and colonic tissue scores. **(C)** Graph of body weight changes in mice and DAI score changes in mice. **(D)** cxcl1; Il-1beta; Il-12; Il-5 and Il-10 between mice with inflammatory bowel disease and healthy mice. DAI score was calculated by a 0–4 scale based on the following parameters: body weight loss (1, 1%−5%; 2, 5%−10%; 3, 10%−15%; and 4, ≥15%), stool consistency (0, normal; 2, loose stools; 4, diarrhea) and blood in the stool (0, no blood seen; 2, apparent blood with stool; 4, grossly bloody stool). Body weight change was calculated by subtracting the initial body weight from the final body weight of the mice. *P < 0.05; **P < 0.01; ***P < 0.001; ****P < 0.0001.

We administered different treatments through the drinking water to three groups of mice, totaling 12 mice (Figure 5A). At the final experimental endpoint, significant differences were observed. Comparison of the colon histopathology of the three groups of mice demonstrated that the M16V-treated colitis mouse model exhibited milder manifestations of colitis, with less infiltration of inflammatory cells, thus suggesting a positive effect of M16V on colitis according to tissue scoring (Figure 5B). We recorded the trend of body weight changes in the three groups of mice and performed significance tests on the differences between the groups. Furthermore, we calculated DAI scores for the three groups of mice to evaluate the severity of colitis. Moreover, the DAI decreased after treatment with M16V, thus indicating that M16V supplementation improved intestinal inflammation (Figure 5C). Quantitative analysis of ten factors demonstrated that among them, five inflammatory factors (CXCL1, IL-1 beta, IL-12, IL-5, and IL-10) exhibited a decreasing trend after the administration of M16V. This finding suggested that M16V can alleviate colitis by inhibiting inflammation (Figure 5D). It provides scientific evidence for the further clinical application of *Bifidobacterium* M16V in the treatment of IBD.

## 4 Discussion

### 4.1 Gut microbiota analysis in pediatric IBD patients

In this investigation, fecal specimens from three distinct cohorts of children (including VEO-IBD, LO-IBD, and HC patients) were subjected to comprehensive analysis to elucidate alterations in the gut microbiota. Our findings underscore a spectrum of intestinal microecological perturbations evident in pediatric patients with IBD, predominately characterized by a decrease in microbial abundance and diversity. This observation aligns with prevailing research (Li H. et al., 2022) and is accompanied by a notable reduction in beneficial probiotic *Bifidobacterium*, which play pivotal roles in immunomodulation, cytokine homeostasis, and maintenance of mucosal integrity (Belkaid and Harrison, 2017). Notably, these alterations were more conspicuous in the VEO-IBD subgroup. Moreover, our investigation demonstrates discernible dissimilarities and heightened heterogeneity in the microbial profiles of children harboring mutations in the IL10RA gene; this suggests a potential exacerbation of intestinal microecological imbalances attributable to this genetic variation. Specifically, the prevalence of *Bifidobacterium*, a key microbial genus, was significantly lower in the VEO-IBD group compared to the other groups. This finding indicates a possible association between *Bifidobacterium* abundance and the pathogenesis of IBD, suggesting that its reduction could have profound implications for disease progression. Furthermore, our study uncovered increased heterogeneity within the microbial profiles of children with IL10RA gene mutations, suggesting that genetic variations may further exacerbate intestinal microecological imbalances.

### 4.2 The role of genetic and microbial factors in the pathogenesis of IBD

Building on our findings, we explored the complex relationship between genetic factors and IBD. Children with early-onset IBD often exhibit more severe symptoms and a higher dependence on immunosuppressive drugs (Abraham et al., 2012). Previous studies have shown that IBD is closely related to genetic factors, with over 200 loci of variation being identified as linked to disease development (Uhlig, 2013). IL10 plays a key role in IL10-mediated signaling to control intestinal inflammation. A deficiency of IL10 and its receptor can lead to impaired signaling, which correspondingly leads to the development of VEO-IBD and a range of clinical signs and symptoms (Mao et al., 2012; Liu and Anderson, 2014). Recent cohort studies have demonstrated a negative correlation between the prevalence of single-gene pathogenicity in IBD patients and the age of disease onset.

The interrelationships between the microbiota, genetic factors, and environmental factors in the pathogenesis of IBD are extremely complex. Although a definitive link between classical pathogens and IBD remains elusive, evidence suggests that the commensal microbiota plays a pivotal role in IBD pathogenesis. For instance, studies have demonstrated that *Aspergillus* can induce colitis in IL10-deficient mice fed a high-fat diet (Devkota et al., 2012), thus highlighting the potential influence of specific microbial strains on disease development. Furthermore, changes in the composition of the gut microbiota can lead to alterations in metabolites. Bile salt hydrolases in certain gut bacteria play a key role in bile acid modification, and impairment of their enzyme activity alters bile salt metabolism, thus rendering the bile acid receptor devoid of anti-inflammatory signaling (Duboc et al., 2013; Cai et al., 2022). Additionally, there are also some bacterial metabolism products, such as short-chain fatty acids (SCFAs), that have the capacity to activate colonic epithelial cells within NLRP3 inflammatory vesicles, thereby contributing to the prevention of colitis (Macia et al., 2015). Recent years have seen an increasing emphasis on the complex interactions between genetic determinants and gut microbiota. Authoritative research has established that genetics significantly influence the composition of gut microbial communities (Lopera-Maya et al., 2022; Qin et al., 2022), with studies identifying a robust correlation between specific genes and bacterial abundance (Vich Vila et al., 2018; Bonder et al., 2016). Emerging evidence suggests that genetic factors play a crucial role in shaping the development and composition of the microbiota, thus modulating changes in cell surface glycosylation and thereby influencing gut microbes (Goodrich et al., 2016; Kudelka et al., 2020).

Our research revealed a heightened heterogeneity of microbiota in children with IL10RA gene mutations, indicating the potential impact of genetic factors on gut microbiota composition. These findings emphasize the impact of gene-microbiota interactions on IBD development. Furthermore, our study demonstrates that the genus *Bifidobacterium* is significantly underrepresented in VEO-IBD. Investigations into the correlation between microbiota and clinical parameters indicate that inflammatory markers, including CRP, ESR and DAI, are negatively correlated with *Bifidobacterium*. This suggests that probiotic interventions may offer promising therapeutic effects to address these microecological imbalances.

### 4.3 Therapeutic potential of probiotics and microbiota modulation

Our research has shown that *Bifidobacterium* are closely related to the calcium signaling pathway. The relationship between the calcium signaling pathway and the intestinal mucosal barrier implies that *Bifidobacterium* may be closely associated with gastrointestinal inflammation (Engevik et al., 2019; Owen et al., 2016). Thus, research on *Bifidobacterium* is a field with scientific promise and potential for clinical translation. M16V is one of the classes of *Bifidobacterium* that can be isolated from the gut of infants and has been referred to as an infantile human-residential *Bifidobacterium* in previous studies (Wong et al., 2018). It has been shown to significantly inhibit Th2 and Th17 lymphocyte subsets, thus exerting a positive effect on early intestinal sectionalization. Additionally, there is considerable evidence for its utility in mitigating atopy and protecting preterm infants from neonatal necrotizing enterocolitis (Li N. et al., 2022; Wong et al., 2019). The study found that *Bifidobacterium* systematically altered gut microbiota composition in a Treg-dependent manner. This change enhanced mitochondrial fitness and IL-10-mediated suppressive functions of intestinal Tregs, contributing to colitis improvement (Sun et al., 2020). Our study also suggests that *Bifidobacterium* M16V can alleviate intestinal inflammation in colitis. Other probiotics, such as *Bacillus subtilis*, have been shown to stimulate T-cell induction, while butyrate-producing species can bolster (Geirnaert et al., 2017; Atarashi et al., 2011). These findings have provided an avenue for innovative therapeutic approaches aimed at harnessing the therapeutic potential of the gut microbiota. Proposed interventions include the administration of multistrain probiotics to ameliorate colitis in UC patients (Bjarnason et al., 2019), as well as the utilization of genetically modified *Escherichia coli* strains to enhance intestinal barrier function and confer resistance against colitis. There are still some risks associated with microbial therapies, such as probiotic bacteremia and the transfer of antibiotic resistance genes to the genomes of pathogenic microorganisms (Nawaz et al., 2011; Arpi et al., 2009). Therefore, the exploration of microbial therapies remains a promising avenue for novel therapeutic strategies in the management of IBD (Praveschotinunt et al., 2019), thus warranting continued investigation and clinical consideration.

### 4.4 Study limitations and future directions

Our study had several limitations as an initial effort to develop a potential modality for microbial therapy. First, the study did not clarify the causal inference between imbalances in the microbiota and IBD, and *Bifidobacterium* M16V was not applied to the population to demonstrate its efficacy. Future randomized, double-blind clinical trials with wider patient populations and longer follow-up times are needed to assess its safety and efficacy. Second, 16S rRNA has limitations, and further studies may be needed to identify functional or metabolic changes in the microbiota. Finally, we have not yet elucidated the exact mechanism of *Bifidobacterium* in the progression of IBD; moreover, similar to other flora, *Bifidobacterium* may be involved in the development of disease through immune regulation, metabolite effects, and the modulation of inflammatory factors. Our study in an animal model provides a basis for its modulation of inflammatory factor release that is expected to provide a new direction for future research, which is a direction of our subsequent research that needs to be refined.

## Data Availability

The datasets presented in this study can be found in online repositories. The names of the repository/repositories and accession number(s) can be found in the article/supplementary material.

## References

[B1] Abbas-Egbariya H. Haberman Y. Braun T. Hadar R. Denson L. Gal-Mor O.. (2022). Meta-analysis defines predominant shared microbial responses in various diseases and a specific inflammatory bowel disease signal. Genome Biol. 23:61. 10.1186/s13059-022-02637-7 35197084 PMC8867743

[B2] Abraham B. P. Mehta S. El-Serag H. B. (2012). Natural history of pediatric-onset inflammatory Bowel disease. J. Clin. Gastroenterol. 46, 581–589. 10.1097/MCG.0b013e318247c32f 22772738 PMC3972042

[B3] Agrawal M. Spencer E. A. Colombel J. F. Ungaro R. C. (2021). Approach to the management of recently diagnosed inflammatory bowel disease patients: a user's guide for adult and pediatric gastroenterologists. Gastroenterology 161, 47–65. 10.1053/j.gastro.2021.04.063 33940007 PMC8640961

[B4] Arnauts K. Sudhakar P. Verstockt S. Lapierre C. Potche S. Caenepeel C.. (2022). Microbiota, not host origin drives *ex vivo* intestinal epithelial responses. Gut Microbes. 14:2089003. 10.1080/19490976.2022.2089003 35758256 PMC9235885

[B5] Arpi M. Vancanneyt M. Swings J. Leisner J. J. (2009). Six cases of *Lactobacillus bacteraemia*: identification of organisms and antibiotic susceptibility and therapy. Scand. J. Infect. Dis. 35, 404–408. 10.1080/00365540310011830 12953954

[B6] Atarashi K. Tanoue T. Shima T. Imaoka A. Kuwahara T. Momose Y.. (2011). Induction of colonic regulatory T cells by indigenous *Clostridium* species. Science 331, 337–341. 10.1126/science.1198469 21205640 PMC3969237

[B7] Belkaid Y. Harrison O. J. (2017). Homeostatic immunity and the microbiota. Immunity 46, 562–576. 10.1016/j.immuni.2017.04.008 28423337 PMC5604871

[B8] Bjarnason I. Sission G. Hayee B. (2019). A randomised, double-blind, placebo-controlled trial of a multi-strain probiotic in patients with asymptomatic ulcerative colitis and Crohn's disease. Inflammopharmacology 27, 465–473. 10.1007/s10787-019-00595-4 31054010 PMC6554453

[B9] Bokulich N. A. Chung J. Battaglia T. Henderson N. Jay M. Li H.. (2016). Antibiotics, birth mode, and diet shape microbiome maturation during early life. Sci. Transl. Med. 8:343ra82. 10.1126/scitranslmed.aad7121 27306664 PMC5308924

[B10] Bonder M. J. Kurilshikov A. Tigchelaar E. F. Mujagic Z. Imhann F. Vila A. V.. (2016). The effect of host genetics on the gut microbiome. Nat. Genet. 48, 1407–1412. 10.1038/ng.3663 27694959

[B11] Cai J. Sun L. Gonzalez F. J. (2022). Gut microbiota-derived bile acids in intestinal immunity, inflammation, and tumorigenesis. Cell Host Microbe. 30, 289–300. 10.1016/j.chom.2022.02.004 35271802 PMC8923532

[B12] Capital Institute of Pediatrics The Coordinating Study Group of Nine Cities on the Physical Growth and Development of Children. (2018). A national survey on physical growth and development of children under seven years of age in nine cities of China in 2015. Zhonghua Er Ke Za Zhi. 56, 192–199. 10.3760/cma.j.issn.0578-1310.2018.03.008 29518829

[B13] Devkota S. Wang Y. Musch M. W. Leone V. Fehlner-Peach H. Nadimpalli A.. (2012). Dietary-fat-induced taurocholic acid promotes pathobiont expansion and colitis in Il10-/- mice. Nature 487, 104–108. 10.1038/nature11225 22722865 PMC3393783

[B14] Duboc H. Rajca S. Rainteau D. Benarous D. Maubert M.-A. Quervain E.. (2013). Connecting dysbiosis, bile-acid dysmetabolism and gut inflammation in inflammatory bowel diseases. Gut 62, 531–539. 10.1136/gutjnl-2012-302578 22993202

[B15] Ekstedt N. Jamioł-Milc D. Pieczyńska J. (2024). Importance of gut microbiota in patients with inflammatory Bowel disease. Nutrients 16:2092. 10.3390/nu16132092 38999840 PMC11242987

[B16] Engevik M. A. Luk B. Chang-Graham A. L. Hall A. Herrmann B. Ruan W.. (2019). Bifidobacterium dentium fortifies the intestinal mucus layer via autophagy and calcium signaling pathways. mBio 10:e01087-19. 10.1128/mBio.01087-19 31213556 PMC6581858

[B17] Geirnaert A. Calatayud M. Grootaert C. Laukens D. Devriese S. Smagghe G.. (2017). Butyrate-producing bacteria supplemented *in vitro* to Crohn's disease patient microbiota increased butyrate production and enhanced intestinal epithelial barrier integrity. Sci. Rep. 7:11450. 10.1038/s41598-017-11734-8 28904372 PMC5597586

[B18] Goodrich J. K. Davenport E. R. Beaumont M. Jackson M. A. Knight R. Ober C.. (2016). Genetic determinants of the gut microbiome in UK twins. Cell Host Microbe. 19, 731–743. 10.1016/j.chom.2016.04.017 27173935 PMC4915943

[B19] Haberman Y. Tickle T. L. Dexheimer P. J. Kim M.-O. Tang D. Karns R.. (2014). Pediatric Crohn disease patients exhibit specific ileal transcriptome and microbiome signature. J. Clin. Invest. 124, 3617–3633. 10.1172/JCI75436 25003194 PMC4109533

[B20] Hall C. H. T. de Zoeten E. F. (2023). Understanding very early onset inflammatory bowel disease (VEOIBD) in relation to inborn errors of immunity. Immunol. Rev. 322, 329–338. 10.1111/imr.13302 38115672 PMC11044353

[B21] Huang Z. Peng K. Li X. Zhao R. You J. Cheng X.. (2017). Mutations in interleukin-10 receptor and clinical phenotypes in patients with very early onset inflammatory bowel disease. Inflamm. Bowel Dis. 23, 578–590. 10.1097/MIB.0000000000001058 28267044

[B22] Hyams J. S. Mandel F. Ferry G. D. Gryboski J. D. Kibort P. M. Kirschner B. S.. (1992). Relationship of common laboratory parameters to the activity of Crohn's disease in children. J. Pediatr. Gastroenterol. Nutr. 14, 216–222. 10.1002/j.1536-4801.1992.tb10528.x 1593378

[B23] IBD Working Group of the European Society for Paediatric Gastroenterology Hepatology and Nutrition. (2005). Inflammatory Bowel disease in children and adolescents: recommendations for diagnosis-the Porto criteria. J. Pediatr. Gastroenterol. Nutr. 41, 1–7. 10.1097/01.MPG.0000163736.30261.82 15990620

[B24] Khalili H. Chan S. S. M. Lochhead P. Ananthakrishnan A. N. Hart A. R. Chan A. T.. (2018). The role of diet in the aetiopathogenesis of inflammatory bowel disease. Nat. Rev. Gastroenterol. Hepatol. 15, 525–535. 10.1038/s41575-018-0022-9 29789682 PMC6397648

[B25] Kostic A. D. Xavier R. J. Gevers D. (2014). The microbiome in inflammatory Bowel disease: current status and the future ahead. Gastroenterology 146, 1489–1499. 10.1053/j.gastro.2014.02.009 24560869 PMC4034132

[B26] Kudelka M. R. Stowell S. R. Cummings R. D. Neish A. S. (2020). Intestinal epithelial glycosylation in homeostasis and gut microbiota interactions in IBD. Nat. Rev. Gastroenterol. Hepatol. 17, 597–617. 10.1038/s41575-020-0331-7 32710014 PMC8211394

[B27] Kuenzig M. E. Fung S. G. Marderfeld L. Mak J. W. Y. Kaplan G. G. Ng S. C.. (2022). Twenty-first century trends in the global epidemiology of pediatric-onset inflammatory bowel disease: systematic review. Gastroenterology 162, 1147–1159.e4. 10.1053/j.gastro.2021.12.282 34995526

[B28] Li H. Wang Y. Shao S. Yu H. Wang D. Li C.. (2022). Rabdosia serra alleviates dextran sulfate sodium salt-induced colitis in mice through anti-inflammation, regulating Th17/Treg balance, maintaining intestinal barrier integrity, and modulating gut microbiota. J. Pharm Anal. 12, 824–838. 10.1016/j.jpha.2022.08.001 36605573 PMC9805946

[B29] Li N. Wang J. Liu P. Li J. Xu C. (2022). Multi-omics reveals that Bifidobacterium breve M-16V may alleviate the immune dysregulation caused by nanopolystyrene. Environ. Int. 163:107191. 10.1016/j.envint.2022.107191 35325770

[B30] Liu J. Z. Anderson C. A. (2014). Genetic studies of Crohn's disease: past, present and future. Best Pract. Res. Clin. Gastroenterol. 28, 373–386. 10.1016/j.bpg.2014.04.009 24913378 PMC4075408

[B31] Lloyd-Price J. Arze C. Ananthakrishnan A. N. Schirmer M. Avila-Pacheco J. Poon T. W.. (2019). Multi-omics of the gut microbial ecosystem in inflammatory bowel diseases. Nature 569, 655–662. 10.1038/s41586-019-1237-9 31142855 PMC6650278

[B32] Lopera-Maya E. A. Kurilshikov A. van der Graaf A. Hu S. Andreu-Sánchez S. Chen L.. (2022). Effect of host genetics on the gut microbiome in 7,738 participants of the Dutch Microbiome Project. Nat. Genet. 54, 143–151. 10.1038/s41588-021-00992-y 35115690

[B33] Macia L. Tan J. Vieira A. T. Leach K. Stanley D. Luong S.. (2015). Metabolite-sensing receptors GPR43 and GPR109A facilitate dietary fibre-induced gut homeostasis through regulation of the inflammasome. Nat. Commun. 6:6734. 10.1038/ncomms7734 25828455

[B34] Mao H. Yang W. Lee P. P. W. Ho M. H. K. Yang J. Zeng S.. (2012). Exome sequencing identifies novel compound heterozygous mutations of IL-10 receptor 1 in neonatal-onset Crohn's disease. Genes Immun. 13, 437–442. 10.1038/gene.2012.8 22476154

[B35] Marteau P. R. Vrese M. D. Cellier C. J. Schrezenmeir J. (2001). Protection from gastrointestinal diseases with the use of probiotics. Am. J. Clin. Nutr. 73, 430s−436s. 10.1093/ajcn/73.2.430s 11157353

[B36] Nawaz M. Wang J. Zhou A. Ma C. Wu X. Moore J. E.. (2011). Characterization and transfer of antibiotic resistance in lactic acid bacteria from fermented food products. Curr. Microbiol. 62, 1081–1089. 10.1007/s00284-010-9856-2 21212956

[B37] Ng S. C. Shi H. Y. Hamidi N. Underwood F. E. Tang W. Benchimol E. I.. (2017). Worldwide incidence and prevalence of inflammatory bowel disease in the 21st century: a systematic review of population-based studies. Lancet 390, 2769–2778. 10.1016/S0140-6736(17)32448-0 29050646

[B38] Nishida A. Inoue R. Inatomi O. Bamba S. Naito Y. Andoh A.. (2017). Gut microbiota in the pathogenesis of inflammatory bowel disease. Clin. J. Gastroenterol. 11, 1–10. 10.1007/s12328-017-0813-5 29285689

[B39] Olbjørn C. Cvancarova Småstuen M. Thiis-Evensen E. Nakstad B. Vatn M. H. Jahnsen J.. (2019). Fecal microbiota profiles in treatment-naive pediatric inflammatory bowel disease-associations with disease phenotype, treatment, and outcome. Clin. Exp. Gastroenterol. 12, 37–49. 10.2147/CEG.S186235 30774408 PMC6362922

[B40] Oliveira S. B. Monteiro I. M. (2017). Diagnosis and management of inflammatory bowel disease in children. BMJ. 357:j2083. 10.1136/bmj.j2083 28566467 PMC6888256

[B41] Owen J. L. Cheng S. X. Ge Y. Sahay B. Mohamadzadeh M. (2016). The role of the calcium-sensing receptor in gastrointestinal inflammation. Semin. Cell Dev. Biol. 49, 44–51. 10.1016/j.semcdb.2015.10.040 26709005 PMC4761506

[B42] Peloquin J. M. Goel G. Villablanca E. J. Xavier R. J. (2016). Mechanisms of pediatric inflammatory bowel disease. Annu. Rev. Immunol. 34, 31–64. 10.1146/annurev-immunol-032414-112151 27168239

[B43] Praveschotinunt P. Duraj-Thatte A. M. Gelfat I. Bahl F. Chou D. B. Joshi N. S.. (2019). Engineered *E. coli* Nissle 1917 for the delivery of matrix-tethered therapeutic domains to the gut. Nat. Commun. 10:5580. 10.1038/s41467-019-13336-6 31811125 PMC6898321

[B44] Qin Y. Havulinna A. S. Liu Y. Jousilahti P. Ritchie S. C. Tokolyi A.. (2022). Combined effects of host genetics and diet on human gut microbiota and incident disease in a single population cohort. Nat. Genet. 54, 134–142. 10.1038/s41588-021-00991-z 35115689 PMC9883041

[B45] Robin X. Turck N. Hainard A. Tiberti N. Lisacek F. Sanchez J. C.. (2011). pROC: an open-source package for R and S+ to analyze and compare ROC curves. BMC Bioinformatics 12;77. 10.1186/1471-2105-12-77 21414208 PMC3068975

[B46] Sharifinejad N. Zaki-Dizaji M. Sepahvandi R. Fayyaz F. Dos Santos Vilela M. M. ElGhazali G.. (2022). The clinical, molecular, and therapeutic features of patients with IL10/IL10R deficiency: a systematic review. Clin. Exp. Immunol. 208, 281–91. 10.1093/cei/uxac040 35481870 PMC9226142

[B47] Subramanian S. Blanton L. V. Frese Steven A. Charbonneau M. Mills David. A Gordon Jeffrey I. (2015). Cultivating healthy growth and nutrition through the gut microbiota. Cell 161, 36–48. 10.1016/j.cell.2015.03.013 25815983 PMC4440586

[B48] Sun S. Luo L. Liang W. Yin Q. Guo J. Rush A. M.. (2020). Bifidobacterium alters the gut microbiota and modulates the functional metabolism of T regulatory cells in the context of immune checkpoint blockade. Proc. Natl. Acad. Sci. 117, 27509–27515. 10.1073/pnas.1921223117 33077598 PMC7959554

[B49] Turner D. Hyams J. Markowitz J. Lerer T. Mack D. R. Evans J.. (2009). Appraisal of the pediatric ulcerative colitis activity index (PUCAI). Inflamm. Bowel Dis. 15, 1218–1223. 10.1002/ibd.20867 19161178

[B50] Uhlig H. H. (2013). Monogenic diseases associated with intestinal inflammation: implications for the understanding of inflammatory bowel disease. Gut 62, 1795–805. 10.1136/gutjnl-2012-303956 24203055

[B51] Uhlig H. H. Charbit-Henrion F. Kotlarz D. Shouval D. S. Schwerd T. Strisciuglio C.. (2021). Clinical genomics for the diagnosis of monogenic forms of inflammatory bowel disease: a position paper from the paediatric IBD porto group of European Society of Paediatric Gastroenterology, Hepatology and Nutrition. J. Pediatr. Gastroenterol. Nutr. 72, 456–473. 10.1097/MPG.0000000000003017 33346580 PMC8221730

[B52] Uhlig H. H. Schwerd T. Koletzko S. Shah N. Kammermeier J. Elkadri A.. (2014). The diagnostic approach to monogenic very early onset inflammatory Bowel disease. Gastroenterology 147, 990–1007.e3. 10.1053/j.gastro.2014.07.023 25058236 PMC5376484

[B53] Vich Vila A. Imhann F. Collij V. Jankipersadsing S. A. Gurry T. Mujagic Z.. (2018). Gut microbiota composition and functional changes in inflammatory bowel disease and irritable bowel syndrome. Sci. Transl. Med. 10:eaap8914. 10.1126/scitranslmed.aap8914 30567928

[B54] Wang H. Vilches-Moure J. G. Cherkaoui S. Tardy I. Alleaume C. Bettinger T.. (2019). Chronic model of inflammatory Bowel disease in IL-10-/- transgenic mice: evaluation with ultrasound molecular imaging. Theranostics 9, 6031–6046. 10.7150/thno.37397 31534535 PMC6735517

[B55] Wang X. Guo L. Huang J. Jiang S. Li N. Mu H. H.. (2023). Plasminogen activator inhibitor-1 potentiates neutrophil infiltration and tissue injury in colitis. Int. J. Biol. Sci. 19, 2132–2149. 10.7150/ijbs.75890 37151884 PMC10158018

[B56] Wang X. Xiao Y. Xu X. Guo L. Yu Y. Li N.. (2021). Characteristics of fecal microbiota and machine learning strategy for fecal invasive biomarkers in pediatric inflammatory Bowel disease. Front. Cell. Infect. Microbiol. 11:711884. 10.3389/fcimb.2021.711884 34950604 PMC8688824

[B57] Wong C. B. Iwabuchi N. Xiao J.-z. (2019). Exploring the science behind *Bifidobacterium breve* M-16V in infant health. Nutrients. 11:1724. 10.3390/nu11081724 31349739 PMC6723912

[B58] Wong C. B. Sugahara H. Odamaki T. Xiao J. Z. (2018). Different physiological properties of human-residential and non-human-residential bifidobacteria in human health. Benef. Microbes. 9, 111–122. 10.3920/BM2017.0031 28969444

[B59] Zegarra Ruiz D. F. Kim D. V. Norwood K. Saldana-Morales F. B. Kim M. Ng C.. (2022). Microbiota manipulation to increase macrophage IL-10 improves colitis and limits colitis-associated colorectal cancer. Gut Microbes 14:2119054. 10.1080/19490976.2022.2119054 36062329 PMC9450902

